# An Environmentally-Adaptive Positioning Method Based on Integration of GPS/DTMB/FM

**DOI:** 10.3390/s18124292

**Published:** 2018-12-06

**Authors:** Li Cong, Haidong Wang, Honglei Qin, Luqi Liu

**Affiliations:** 1 School of Electronic and Information Engineering, Beihang University, No. 37 Xueyuan Road, Haidian District, Beijing 100191, China; congli_bh@buaa.edu.cn (L.C.); whdtune@gmail.com (H.W.); 2 Didi Chuxing Technology Co., Building B1&B2, Digital Valley, Zhongguancun Software Park, Compound 8, Dongbeiwang Road, Haidian District, Beijing 100000, China; liuluqi@didiglobal.com

**Keywords:** Global Positioning System, Digital Terrestrial Multimedia Broadcast, frequency modulation, environmentally adaptive, Fuzzy Inference System, Extended Kalman Filter

## Abstract

The Global Positioning System (GPS) yields good precision and availability in open outdoor environment. However, the errors of GPS may suffer degradation in some complex environments, such as forests and urban canyons. To solve this problem, a new positioning method is designed integrating GPS, Digital Terrestrial Multimedia Broadcast (DTMB) and frequency-modulated (FM) radio signal. In this method, the DTMB transmitter acts as a pseudo-satellite to assist GPS positioning. Furthermore, the FM fingerprint positioning is used to correct the positioning bias. An adaptive selection scheme is proposed to provide an optimal integration mode of the sensors. Field experiments in complex environment were carried out for evaluation. Comparing to the GPS-only and GPS + DTMB approach, positioning accuracy was improved by at least 68.21% and 21.27% with the proposed method, respectively.

## 1. Introduction

After decades of development, the Global Positioning System (GPS) has become the dominant positioning approach in outdoor environment for its high precision, globalization and real-time response. Advances in technology have facilitated the application of smartphones with cheap GPS receivers, providing users with access to location service in common outdoor environment. However, GPS signal may be confronted with attenuation, occlusion and multipath in scenarios such as forests and urban canyons, which often leads to serious deterioration of positioning accuracy, or even failure in positioning. To tackle the positioning problem in such complex environment, various positioning techniques have emerged, such as ultra-wideband (UWB) [1,2], frequency-modulated (FM) radio [3,4,5,6,7,8], digital television (DTV) [9,10,11], radio-frequency identification (RFID) [12], and WiFi [13,14,15,16,17,18]. UWB positioning systems locate users using a trilateration algorithm with the help of pre-deployed UWB transmitters. Although it may achieve high accuracy, the cost of positioning environment setup is expensive. RFID-based positioning systems determine users’ locations when they are in the vicinities of pre-deployed RFID tags. Effective read distance of RFID tag is usually limited, so it would take both time and labor to deploy many tags for the purpose of positioning in vast areas. WiFi positioning, as a popular technique in indoor positioning, is not suitable for outdoor scenarios since it requires good WiFi coverage. As for signal with lower frequency such as DTV and FM signal, mature infrastructure and strong propagation ability make them good choices for positioning in areas short of GPS signal. Thus, DTV and FM signal are employed in this paper to assist GPS signal to achieve positioning in complex environment with GPS failure.

Digital Terrestrial Multimedia Broadcasting (DTMB) is the standard for digital television broadcasting systems in China [19]. At the headers of DTMB signal frames are pseudo-noise sequences, which act as guard intervals and basis of temporal synchronization. By frame synchronization [20,21,22], we can know the signal propagation duration and the distance between user and transmitter [23]. Reference time used by DTMB transmitters is strictly synchronized with GPS time. Thus, it is possible to locate users with the trilateration approach when signals from four or more DTMB transmitters are available [11]. In some regions, it might be difficult to receive signals from more than three DTMB transmitters simultaneously due to transmitter planning reasons. In this case, navigation information from DTMB and GPS, like pseudo-range and pseudo-range rate, can be fused to provide more accurate user positions than either alone [9,24,25]. Signal strength of DTMB signal is usually much stronger than that of GPS signal since the former is broadcasted by fixed ground stations. Thanks to its low frequency of 470–860 MHz, DTMB signal shows stronger penetration and propagation ability than many other signals with higher frequency. Its high availability can also be employed to perform fingerprint positioning [10].

Frequency-modulated (FM) radio broadcasting has long been widely used. Its low frequency, around 100 MHz, makes it much easier to propagate in forests, urban canyons and indoor scenarios than other common signals such as GPS, WiFi and DTMB signal. The propagation model approach and the fingerprinting approach are usually employed in FM-based positioning systems. The propagation model method deduces user locations by analyzing signal characteristics measured by the user with prebuilt radio propagation model. The fingerprinting method [26,27,28] is based on similarity of signal characteristics among locations, whereas the signal characteristics are referred to as “fingerprints” at corresponding locations. Fingerprint positioning is generally divided into two stages: the offline stage and the online stage. In the offline stage, site survey is conducted to collect fingerprint data at reference points (RPs) and create fingerprint database. In the online stage, fingerprint data at test points (TPs) are compared with the database using feature matching methods to estimate users’ positions. The two approaches can also be integrated together to achieve better accuracy [8].

The principle of DTMB positioning in our method is similar to that of GPS, thus it is not difficult for us to aid GPS with DTMB. However, line-of-sight (LOS) propagation of GPS/DTMB signal is required for good precision. On the other hand, FM fingerprint positioning system can work in environment with little LOS signal. Even though the positioning accuracy of FM fingerprinting is not as competitive as that of GPS, it can be improved if GPS is used to constrain the range of RPs. Therefore, we integrate GPS, DTMB and FM signal and adaptively select the integration mode according to environmental conditions to minimize positioning error.

In this paper, an outdoor positioning system combining GPS, DTMB and FM signals is presented to tackle the positioning problem in complex outdoor environment. The proposed system can adaptively select the appropriate integration mode of the three signals by analyzing environmental characteristics, and then fusing information from part or all of the three signals with an Extended Kalman Filter (EKF) to yield the optimal positioning result.

The rest of the paper is organized as follows: Section 2 introduces the overall system architecture. Section 3 describes the details. Section 4 presents the design of the mode selection scheme. Section 5 explains design of the sensor fusion filter. In Section 6, experiments and result analysis are provided. In Section 7, conclusions and future works are summarized.

## 2. System Architecture

**Figure 1:**
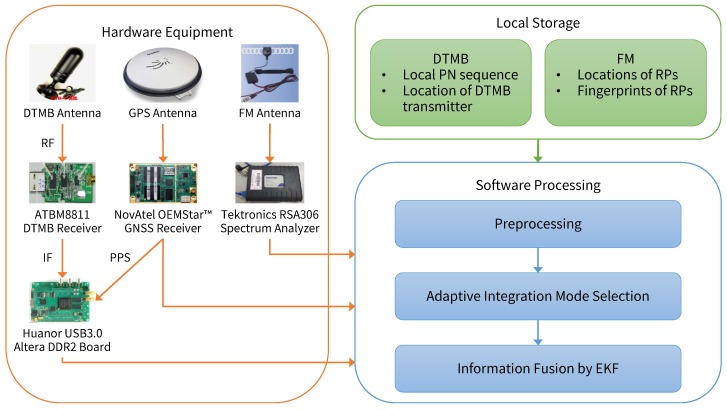
Overall architecture of the proposed system.

The integrated positioning system designed in this paper consists of three parts: hardware equipment, software processing and local storage, as shown in Figure 1. Hardware equipment includes GPS antenna and receiver, DTMB antenna and receiver, and FM antenna and receiver. With the hardware equipment, we can receive and extract raw information from the signals. Software processing is composed of three stages: preprocessing, adaptive integration mode selection and information fusion by EKF. In the preprocessing stage, GPS is employed to assist DTMB and FM according to environmental conditions and input for the integration mode selection stage is computed. In the mode selection stage, a fuzzy inference system is utilized to select the appropriate integration mode of GPS, DTMB and FM signals based on several environmental indicators. In the information fusion stage, information from the three signals are fused by EKF to estimate user locations based on mode selection result. Information needed by software processing, such as DTMB pseudo-noise sequence and FM fingerprint database, is managed by local storage.

## 3. Preprocessing

**Figure 2:**
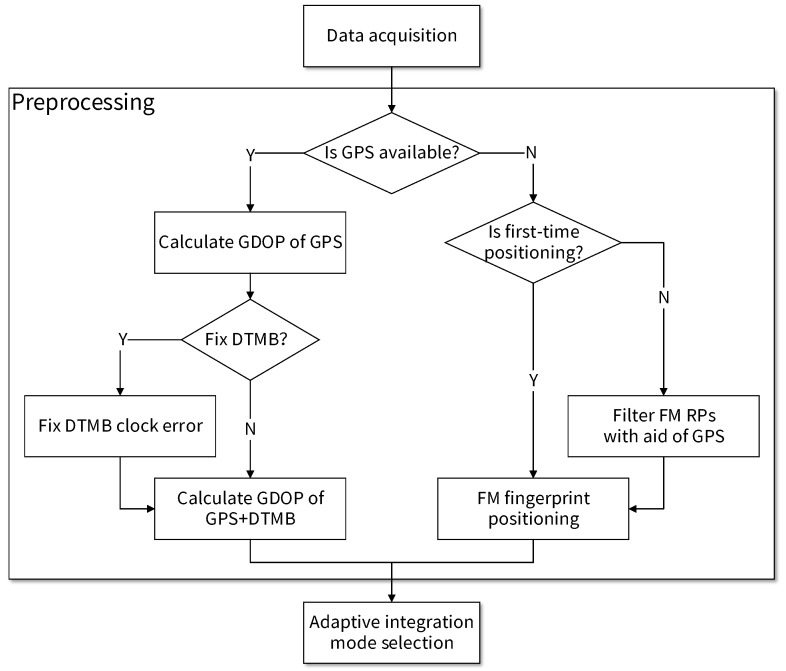
Flow chart of preprocessing.

Software processing is made up of preprocessing, adaptive mode selection and information fusion. Flow chart of the preprocessing part is shown in Figure 2. First, whether GPS positioning result is available is checked by the number of visible satellites. If it is available, the geometric dilution of precision (GDOP) of GPS positioning is calculated and whether we can use GPS to correct clock error of DTMB is checked. Then, if the correction is permitted, it is conducted and GDOP of GPS + DTMB positioning is calculated as input variable for mode selection part. If the number of visible satellites is too few for us to get reliable GPS positioning results, FM fingerprint positioning if performed. If GPS positioning had been performed before, we can exploit previous GPS positioning results to attain an estimation of the current user location, and use it to remove FM RPs that are too far away for better accuracy. Errors of DTMB-only or FM-only positioning are often larger than those of GPS only, thus it is necessary to perform DTMB clock error correction and FM RP selection with GPS data for location accuracy improvement. GDOP of GPS alone, GDOP of GPS + DTMB, and RP number within constraint range are needed in the integration mode selection stage.

### 3.1. DTMB Clock Error Correction

When GDOP of GPS is no greater than a certain threshold, it can be considered that positioning requirement is satisfied and we can use GPS positioning results to correct clock error of the DTMB receiver.

Equation (1) shows the relationship between satellite positions and user location in GPS positioning:

(1) $$\rho_{s}^{i}=\sqrt{{\left(x^{i}-x\right)}^{2}+{\left(y^{i}-y\right)}^{2}+{\left(z^{i}-z\right)}^{2}}+c\cdot \Delta t_{u}+\varepsilon_{\rho }^{i}\;,\;\left(i=1,2,\cdots ,N\right)\;.$$

It reveals the position relationship between user and the *i*th GPS satellite, where $\rho_{s}^{i}$ is the pseudorange of the satellite, $\left[x^{i},y^{i},z^{i}\right]$ is the position of the satellite, [x,y,z] is the user’s location, *c* is the speed of light, $\Delta t_{u}$ is the clock error of GPS receiver, *N* is the number of available GPS satellites and $\varepsilon_{\rho }^{i}$ is the GPS pseudorange measurement noise. Equation (2) shows the position relationship between user and DTMB transmitter:

(2) $$\rho_{d}=\sqrt{{\left(x^{d}-x\right)}^{2}+{\left(y^{d}-y\right)}^{2}+{\left(z^{d}-z\right)}^{2}}+c\cdot \Delta t_{d}+\varepsilon_{d}\;,$$

where $\rho_{d}$ is the pseudorange between user and the transmitter, $\left[x^{d},y^{d},z^{d}\right]$ is the position of the transmitter, $\Delta t_{d}$ is the clock error of DTMB receiver and $\varepsilon_{d}$ is the DTMB pseudorange measurement noise. When there are at least four GPS satellites available, we can obtain user position with Equation (1), after which clock error of DTMB can be attained with Equation (2). In this way, clock error correction for DTMB is performed.

### 3.2. FM RP Selection

**Figure 3:**
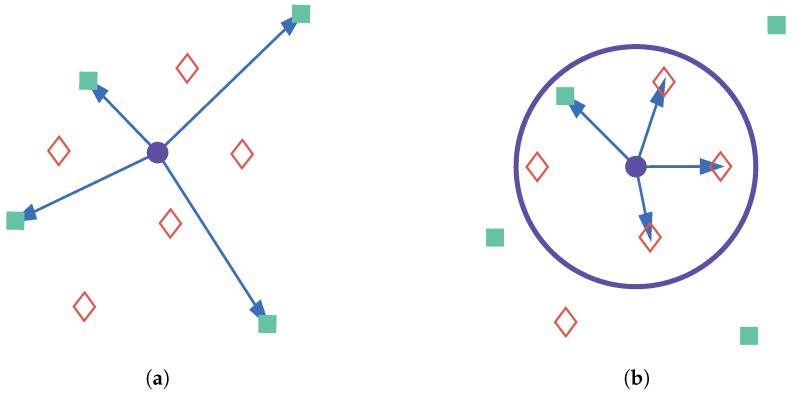
Demonstration of RP constraint: (**a**) RP selection result without GPS constraint; and (**b**) RP selection result with GPS constraint. In (**a**,**b**), the purple circles are TPs. In (**a**), cyan rectangles connected with TP by arrows are selected RPs and red diamonds are RPs not selected for computation of TP’s position. In (**b**), points connected with TP by arrows are selected RPs, the purple circle shows the constraint range.

If GDOP of GPS is small enough, GPS system can also be employed to improve positioning accuracy of FM fingerprinting. The basic assumption for fingerprint localization is that spacial proximity is proportional to similarity of signal characteristics. However, this assumption is not always true in actual conditions. There is the possibility that the RP with the highest similarity in signal characteristics is far from the TP, whereas RPs in the vicinity of the TP demonstrate low similarity with it, as shown in Figure 3a. Therefore, GPS positioning result is utilized to filter out RPs too far from the current TP to reduce such cases. When GPS positioning condition is good enough, we can take the GPS positioning result as the tentative position of TP, and draw a circle on the ground plane with some radius, as illustrated in Figure 3b. To restrict the upper bound of the positioning error, only RPs within this circle can be selected for computation of fingerprinting result. On the other hand, when GPS positioning result is not reliable, we can deduce user’s position with previous reliable GPS positioning results and increase the radius of the circle. This is usually effective when GDOP of GPS has just begun to deteriorate.

## 4. Adaptive Integration Mode Selection

Various factors indicate environment conditions, such as GDOP of GPS, GDOP of GPS + DTMB, and the number of RPs within constraint range for FM. It is not easy to find a specific formula describing the relationship between integration mode and these factors. Thus, we adopt the fuzzy inference approach to combine these factors and find the optimal integration mode of GPS, DTMB and FM signals.

Fuzzy inference system is predicated upon the fuzzy set theory. In the set theory, the relationship between an element and a set is crisp, either belonging to or not belonging to. However, the relationship between an element and a fuzzy set is described by membership ranging from 0 to 1. The crisp inputs are fuzzified into memberships to fuzzy sets, but the final outputs are crisp values after defuzzification.

The inputs of our fuzzy inference system are GDOP of GPS, GDOP of GPS + DTMB, and number of FM RPs within constraint range. The output is the integration mode: GPS only, GPS + DTMB, and GPS + DTMB + FM. First trigonometric membership functions are utilized to fuzzify the inputs and convert them into memberships. Then, we need to create fuzzy rules to connect inputs and outputs. The fuzzy rules in our system can be described as below:

(3) $$\begin{array}{c} \begin{array}{cc} R^{i}:\;\;\;IF & \;x_{1}\subset X_{1}^{a}\;AND\;x_{2}\subset X_{2}^{b}\;AND\;x_{3}\subset X_{3}^{c}\\ THEN & \;y\subset Y^{d}\;,\;a,c=1,2;\;b,d=1,2,3;\;i=1,2,\cdots ,n\;, \end{array} \end{array}$$

where $R^{i}$ is the *i*th rule; $x_{1}$, $x_{2}$, and $x_{3}$ are the fuzzified inputs; $X_{1}^{a}$, $X_{2}^{b}$, and $X_{3}^{c}$ are the fuzzy sets for inputs; *y* is the output before defuzzification; and $Y^{d}$ is the fuzzy set for the output.

**Figure 4:**
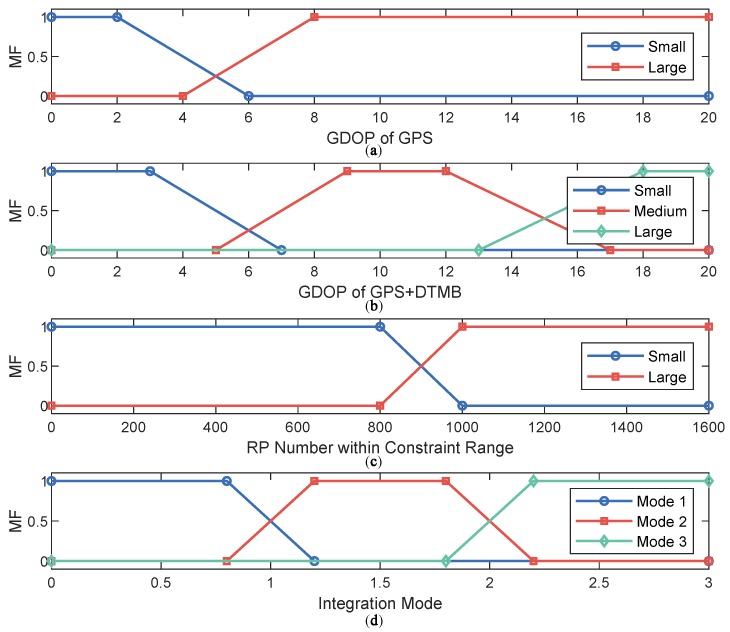
Membership functions in our fuzzy inference system: (**a**) membership function for GDOP of GPS; (**b**) membership function for GDOP of GPS + DTMB; (**c**) membership function for RP number within constraint range; and (**d**) membership function for system output (integration mode).

There are two fuzzy sets for both of the input $x_{1}$ and $x_{3}$ for description of small and large values of them, correspondingly, $X_{1}^{S}$, $X_{1}^{L}$, $X_{2}^{S}$ and $X_{2}^{L}$. For the input $x_{2}$, three fuzzy sets are used, $X_{2}^{S}$, $X_{2}^{M}$ and $X_{2}^{L}$, for description. The three integration mode are denoted by $Y^{A}$, $Y^{B}$ and $Y^{C}$, as shown in Table 1. Membership functions (MF) for inputs and outputs of the fuzzy inference system are shown as Figure 4. In this way, all fuzzy rules are demonstrated, as shown in Table 2.

## 5. Design of Integration Filter

In this paper, EKF is utilized for fusion of information from multiple sources. The design of state model and observation model in our EKF filter is explained in detail below.

### 5.1. State Model

System state in our filter is a vector of 11 dimensions, including position, velocity, acceleration, clock error and frequency error of user’s receiver, as illustrated in Equation (4):

(4) $$x={\left[x,\dot{x},\ddot{x},y,\dot{y},\ddot{y},z,\dot{z},\ddot{z},\delta t,\delta f\right]}^{T}\;,$$

where [x,y,z] is the receiver coordinate in the earth-centered earth-fixed (ECEF) coordinate system, $\left[\dot{x},\dot{y},\dot{z}\right]$ is the receiver velocity in the ECEF coordinate system, $\left[\ddot{x},\ddot{y},\ddot{z}\right]$ is the receiver acceleration in the ECEF coordinate system, δt is the receiver clock error, and δf is the receiver frequency error. In the integrated system, the acquisition of the DTMB signal is triggered by GPS pulses-per-second signal. DTMB clock error can be corrected with the aid of GPS when the latter is ready for use. Therefore, it can be deemed that clock time of the GPS receiver and that of DTMB receiver is approximately synchronized, and the two receivers also share the same clock error and frequency error.

By referring to the established model, the discrete state model of EKF is shown as Equation (5):

(5) $$x_{k+1}=\Phi_{k}\cdot x_{k}+\omega_{k}=[\begin{array}{cccc} M & 0 & 0 & 0\\ 0 & M & 0 & 0\\ 0 & 0 & M & 0\\ 0 & 0 & 0 & A_{c} \end{array}]x_{k}+\omega_{k}\;,$$

where $M=[\begin{array}{ccc} 1 & T & T^{2}/2\\ 0 & 1 & T\\ 0 & 0 & 1 \end{array}]$, $A_{c}=[\begin{array}{cc} 1 & T\\ 0 & 1 \end{array}]$, where *T* is the differential interval and $\omega_{k}$ is the process noise vector. The covariance matrix of $\omega_{k}$, Q, is:

(6) $$Q=[\begin{array}{cc} Q_{p} & 0\\ 0 & Q_{c} \end{array}]\;.$$

In Equation (6), $Q_{p}={\left[Q_{x},Q_{y},Q_{z}\right]}^{T}$, and $Q_{k}\left(k=x,y,z\right)$ is the covariance matrix of the vector ${\left[k,\dot{k},\ddot{k}\right]}^{T}\left(k=x,y,z\right)$. $Q_{k}\left(k=x,y,z\right)$, which can be obtained as Equation (7):

(7) $$Q_{k}=S_{a,k}[\begin{array}{ccc} T^{5}/20 & T^{4}/8 & T^{3}/6\\ T^{4}/8 & T^{3}/3 & T^{2}/2\\ T^{3}/6 & T^{2}/2 & T \end{array}]\;,\;\left(k=x,y,z\right)\;,$$

where $S_{a,k}$ is the power spectral density of the receiver acceleration with respect to the *k* axis. On the other hand, $Q_{C}$ in Equation (6) is the covariance matrix of the vector ${\left[\delta t,\delta f\right]}^{T}$ and it can be attained as Equation (8):

(8) $$Q_{c}=[\begin{array}{cc} S_{t}T+S_{f}T^{3}/3 & S_{f}T^{2}/2\\ S_{f}T^{2}/2 & S_{f}T \end{array}]\;,$$

where $S_{t}$ and $S_{f}$ are the noise power spectral density of the clock error and frequency error.

### 5.2. Observation Model

There are three types of observations in our filter: pseudoranges obtained from GPS and DTMB, pseudorange rate from GPS, and position coordinate from FM.

#### 5.2.1. Pseudorange Observation

The pseudorange of the *i*th satellite, $\rho^{i}$, can be expressed as below:

(9) $$\rho^{i}=h_{s}\left(x\right)={\left[{\left(x_{s}^{i}-x\right)}^{2}+{\left(y_{s}^{i}-y\right)}^{2}+{\left(z_{s}^{i}-z\right)}^{2}\right]}^{1/2}+\Delta t+\varepsilon_{\rho }^{i}\;,\;\left(i=1,2,\cdots ,N\right)\;,$$

where *N* is the total number of satellites, $\left[x_{s}^{i},y_{s}^{i},z_{s}^{i}\right]$ is the satellite position in the ECEF coordinate system calculated from GPS ephemeris and $\varepsilon_{\rho }^{i}$ is measurement noise of the pseudorange. The partial derivatives of $h_{s}\left(x\right)$ are as follows:

(10) $$\begin{array}{cc} \frac{\partial h_{s}}{\partial x} & =-\frac{x_{s}^{i}-\hat{x}}{{\left[{\left(x_{s}^{i}-\hat{x}\right)}^{2}+{\left(y_{s}^{i}-\hat{y}\right)}^{2}+{\left(z_{s}^{i}-\hat{z}\right)}^{2}\right]}^{1/2}}\\ & =\frac{\hat{x}-x_{s}^{i}}{r^{i}}=e_{1}^{i}\;, \end{array}$$

(11) $$\frac{\partial h_{s}}{\partial y}=\frac{\hat{y}-y_{s}^{i}}{r^{i}}=e_{2}^{i}\;,$$

(12) $$\frac{\partial h_{s}}{\partial z}=\frac{\hat{z}-z_{s}^{i}}{r^{i}}=e_{3}^{i}\;.$$

In Equations (10)–(12), $e_{j}^{i}\left(i=1,2,\cdots ,N;j=1,2,3\right)$ denotes the partial derivative $\partial h_{s}/\partial k\left(k=x,y,z\right)$ and $r^{i}\left(i=1,2,\cdots ,N\right)$ is the distance between user and the *i*th satellite. Thus, assuming the number of visible satellites is *m*, the Jacobian matrix of $h_{s}\left(x\right)$ is shown as Equation (13):

(13) $$H_{\rho ,s}=[\begin{array}{ccccccccccc} e_{11} & 0 & 0 & e_{12} & 0 & 0 & e_{13} & 0 & 0 & 1 & 0\\ e_{21} & 0 & 0 & e_{22} & 0 & 0 & e_{23} & 0 & 0 & 1 & 0\\ \vdots & \vdots & \vdots & \vdots & \vdots & \vdots & \vdots & \vdots & \vdots & \vdots & \vdots \\ e_{m1} & 0 & 0 & e_{m2} & 0 & 0 & e_{m3} & 0 & 0 & 1 & 0 \end{array}]\;.$$

Similarly, the pseudorange of DTMB is shown as below:

(14) $$\rho_{d}=h_{d}\left(x\right)={\left[{\left(x_{d}-x\right)}^{2}+{\left(y_{d}-y\right)}^{2}+{\left(z_{d}-z\right)}^{2}\right]}^{1/2}+\Delta t+\varepsilon_{\rho }\;,$$

where ${\left[x_{d},y_{d},z_{d}\right]}^{T}$ is the position of the DTMB transmitter in the ECEF coordinate system and $\varepsilon_{\rho }$ is the measurement noise of the DTMB pseudorange. Now, we can get a Jacobian matrix for the pseudorange:

(15) $$H_{\rho ,d}=[\begin{array}{ccccccccccc} (\hat{x}-x_{d})/r & 0 & 0 & (\hat{y}-y_{d})/r & 0 & 0 & (\hat{z}-z_{d})/r & 0 & 0 & 1 & 0 \end{array}]\;.$$

From the above, we can know the observation model is the same for the pseudorange of both GPS and DTMB. Therefore, the DTMB transmitter is taken as a pseudo satellite in our algorithm and Equation (9) is the uniform observation equation when pseudorange is used as the observation.

#### 5.2.2. Pseudorange Rate Observation

From Equation (9), we can get the observation equation when pseudorange rate is employed as observation:

(16) $${\dot{\rho }}_{s}^{i}=\frac{\partial \rho_{s}^{i}}{\partial t}=e_{1}^{i}\left({\dot{x}}_{s}^{i}-\dot{x}\right)+e_{2}^{i}\left({\dot{y}}_{s}^{i}-\dot{y}\right)+e_{3}^{i}\left({\dot{z}}_{s}^{i}-\dot{z}\right)+\Delta f+\varepsilon_{\dot{\rho }}^{i}\;,\;\left(i=1,2,\cdots ,N\right)\;,$$

where ${\left[{\dot{x}}_{s}^{i},{\dot{y}}_{s}^{i},{\dot{z}}_{s}^{i}\right]}^{T}$ is the satellite velocity in ECEF coordinate system computed from satellite ephemeris and $\varepsilon_{\dot{\rho }}^{i}$ is measurement noise of the pseudorange rate. The corresponding Jacobian matrix is:

(17) $$H_{\dot{\rho },s}=[\begin{array}{ccccccccccc} 0 & e_{11} & 0 & 0 & e_{12} & 0 & 0 & e_{13} & 0 & 0 & 1\\ 0 & e_{21} & 0 & 0 & e_{22} & 0 & 0 & e_{23} & 0 & 0 & 1\\ \vdots & \vdots & \vdots & \vdots & \vdots & \vdots & \vdots & \vdots & \vdots & \vdots & \vdots \\ 0 & e_{m1} & 0 & 0 & e_{m2} & 0 & 0 & e_{m3} & 0 & 0 & 1 \end{array}]\;.$$

#### 5.2.3. Position Observation

Position information can be obtained from FM fingerprint positioning. When we use the FM signal to correct state prediction, position $L_{f}={\left[x_{f},y_{f},z_{f}\right]}^{T}$ from FM fingerprinting is used as the observation. The corresponding observation equation is shown as follows:

(18) $$L_{f}=[\begin{array}{c} x_{f}\\ y_{f}\\ z_{f} \end{array}]+[\begin{array}{c} \varepsilon_{x}\\ \varepsilon_{y}\\ \varepsilon_{z} \end{array}]=H_{f}x_{k}+\varepsilon_{f}\;,$$

where $H_{f}={\left[diag\left[1\;1\;1\right]\;0_{3\times 8}\right]}_{3\times 11}$ and $\varepsilon_{f}$ is the measurement noise of position. The covariance matrix of $\varepsilon_{f}$ is:

(19) $$R_{f}=E\left[\varepsilon_{f}\varepsilon_{f}^{T}\right]=[\begin{array}{ccc} \sigma_{x,f}^{2} & & \\ & \sigma_{y,f}^{2} & \\ & & \sigma_{x,z}^{2} \end{array}]\;,$$

where $\sigma_{x,f}^{2}$, $\sigma_{y,f}^{2}$ and $\sigma_{z,f}^{2}$ are the positioning error variances of the FM fingerprinting method with respect to the *x*, *y* and *z* directions.

## 6. Experiments and Analysis

The proposed algorithm was tested at two sites, one on the campus of Beihang University and the other on Kehui Road of Beijing.

**Figure 5:**
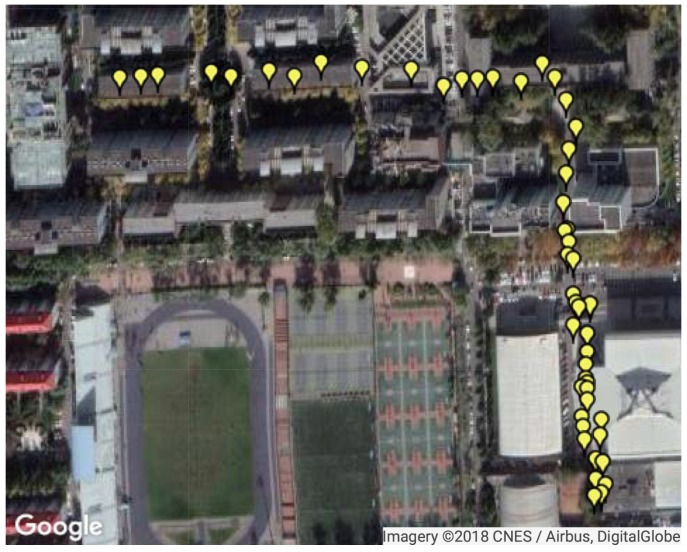
The first test site and test points. The yellow markers on the map denote the test points.

The first test region in Beihang University covers an area of around 380 m × 380 m, as shown in Figure 5. Fifty points were chosen as TP and 282 as RP for the data collection. Then, Kriging interpolation was applied to our collected RP fingerprints to increase the reference point density in a grid style. After interpolation, there were 16,640 RPs in total and the minimum distance between two RPs was 3 m. All of the GPS, DTMB and FM signals were received and measured at the test points, whereas only the FM signal was measured at the reference points. For GPS, the signal used by us is GPS L1 signal. For DTMB, it is DTMB Channel 14 in Beijing. As for FM radio, 21 channels with strong signal strength in Beijing were selected. True locations of the points were obtained using a Novatel IMU-FSAS inertial measurement unit. 

**Figure 6:**
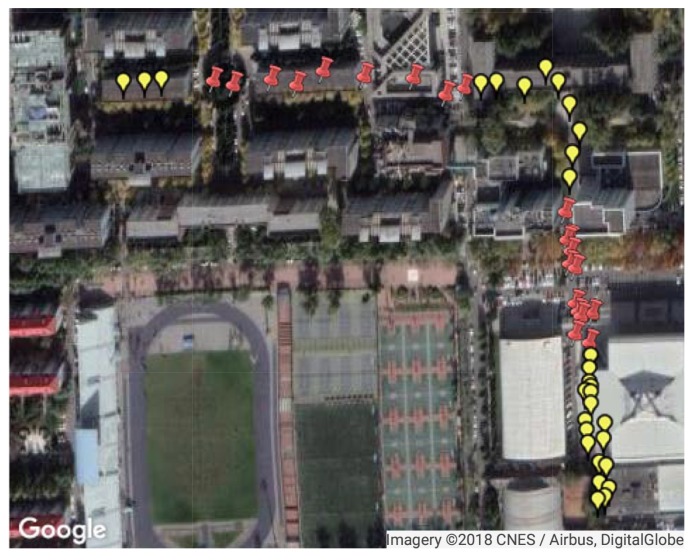
Illustration of points with and without manual satellite loss at Site 1. The red pushpins are points with satellite loss.

**Figure 7:**
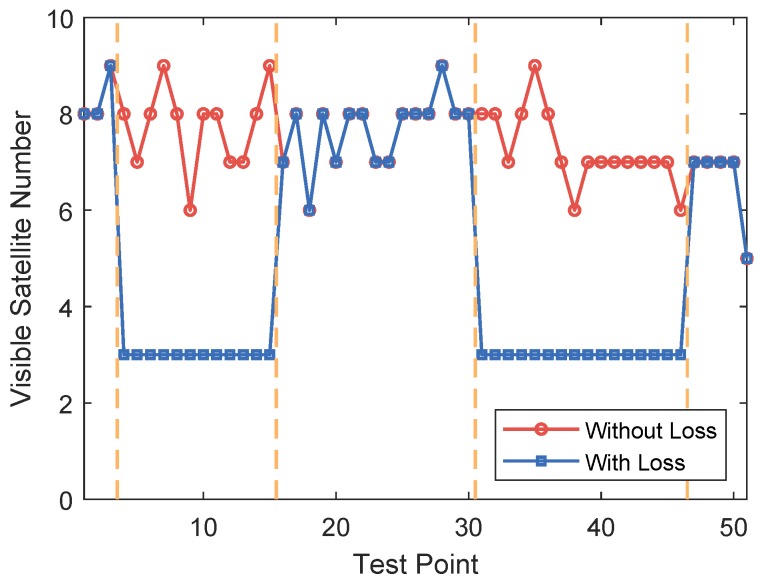
Number of available satellites at each test point at Site 1.

**Figure 8:**
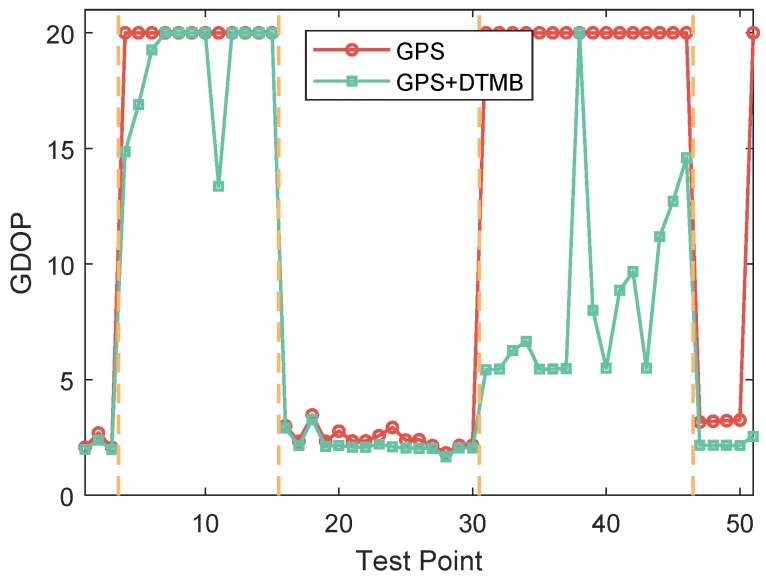
GDOP of GPS and GPS + DTMB at each test point at Site 1.

Since the availability of GPS satellites is rather good (no fewer than four satellites available at each TP) at the two test areas, loss of satellite signal was achieved by manually shielding some of the satellites. Two subareas were chosen as area with satellite loss, where available satellite number was restricted to three. For simplification of expression, the 50 TPs were numbered from 1 to 50. TPs numbered from 4 to 15 and from 31 to 46 are test points with satellite loss, as shown in Figure 6. The numbers of available satellites at each point before and after manual shielding are shown in Figure 7. The vertical dashed lines in the figure denote the boundaries between the areas with and without satellite loss. In the figure, we can see that at each point there are at least five satellites available without the satellite loss. The GDOPs of each point under GPS-only and GPS + DTMB mode are illustrated in Figure 8. Here, we manually set GDOP to 20 when it exceeds 20 for convenience in figure plotting. Putting Figure 7 and Figure 8 together, it can be seen that there are at least six available satellites without satellite loss and GDOP of GPS-only mode is no greater than 5. When the number of available satellites is reduced to three, GDOP of GPS-only mode spikes to 20. However, once DTMB was introduced to work together with GPS, GDOP stays below that of GPS-only most of the time, especially in the case of satellite loss. Therefore, it can be concluded that DTMB can improve satellite geometric distribution when the availability of GPS satellites is poor. 

**Figure 9:**
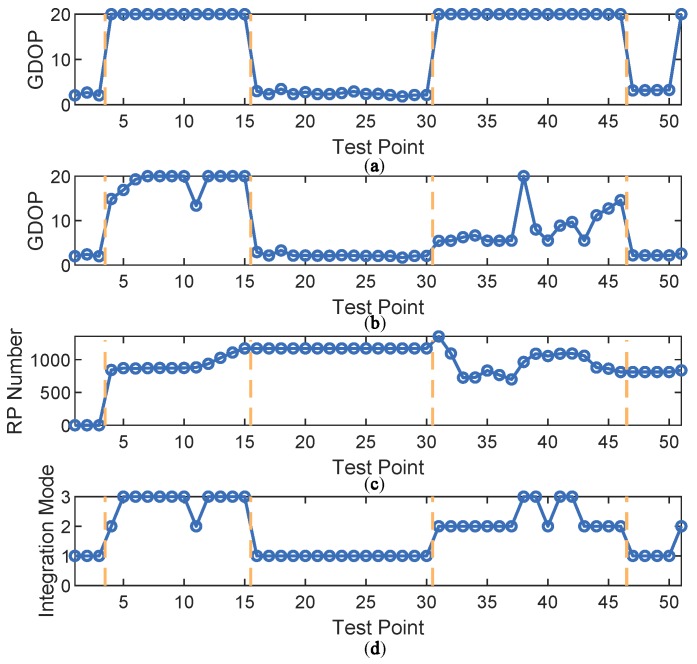
The inputs and output of the fuzzy inference system at Site 1: (**a**) GDOP of GPS-only mode; (**b**) GDOP of GPS + DTMB; (**c**) the number of RPs within constraint range; and (**d**) integration mode.

Then, the proposed fuzzy inference system was applied to determine the optimal integration mode at each of the test points. The inputs and output of the fuzzy system are shown in Figure 9. The test points were divided into five sections by the four vertical dashed lines. For points indexed 1, 2 and 3 in the first section, GPS-only positioning error stayed low with sufficient available satellites, leading to choice of the GPS-only mode. In the second section, the GPS-only mode was no longer chosen because GDOP of GPS-only mode increased due to satellite loss. At this time, GDOP of GPS + DTMB varied with time. When GDOP of GPS + DTMB is relatively small, this mode is preferred. However, if it is too large, FM will be introduced to assist them, which is the GPS + DTMB + FM mode. In the third section, available satellite number became adequate again and GPS-only mode was chosen. In the fourth section, GDOP of GPS + DTMB was relatively small most of the time, resulting in the choice of the GPS + DTMB mode. Under some circumstances where GDOP of GPS + DTMB is not small enough and RP number within constraint range is sufficient, the GPS + DTMB + FM mode can yield more precise positioning results. In the last section, GPS-only mode was selected when there was enough available satellites. In other cases, where GDOP of GPS is too large, the GPS + DTMB mode is favored as GDOP is limited to a small level with the help of DTMB.

**Figure 10:**
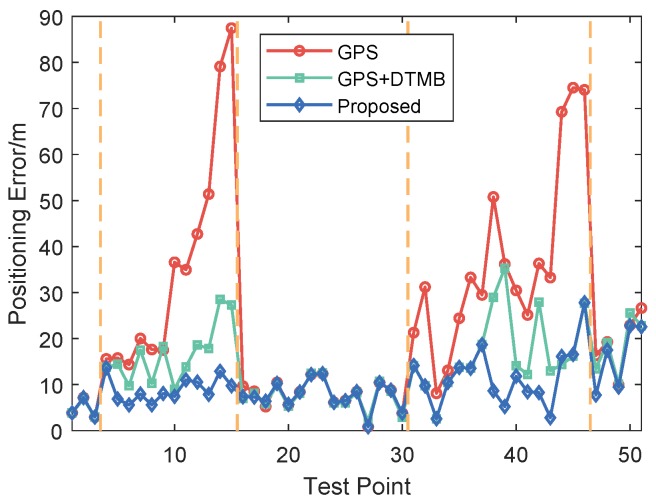
Positioning error at each test point at Site 1.

**Figure 11:**
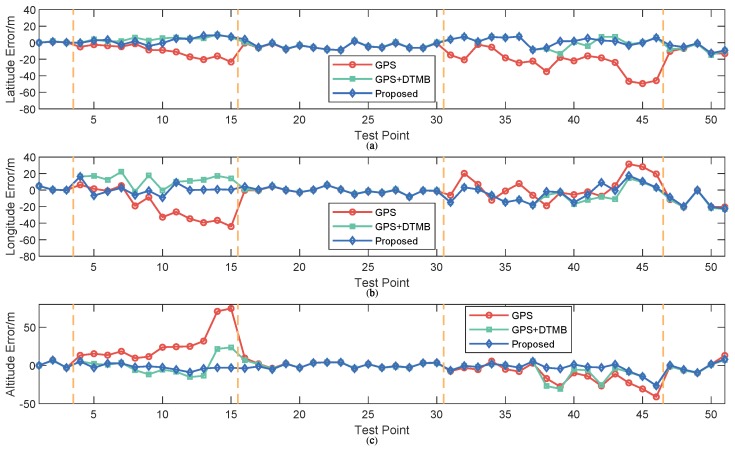
Positioning error of each point in latitude/longitude/altitude direction. (**a**) error in latitude direction; (**b**) error in longitude direction; and (**c**) error in altitude direction.

**Figure 12:**
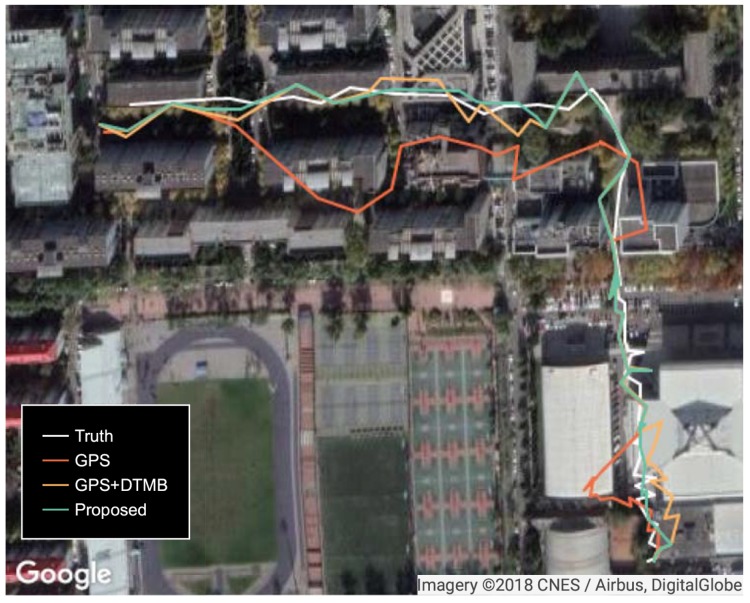
Trajectories generated from true positions and positioning results at Site 1.

To show the performance of the proposed approach, positioning error of the proposed method was compared with that of GPS and GPS + DTMB. Positioning error of each test point is shown in Figure 10, where it is demonstrated that overall positioning accuracy of the proposed method outperformws the other two, and the incorporation of DTMB reduced positioning error of GPS. Errors of the three methods in latitude/longitude/altitude direction are also provided (Figure 11), where we found that the proposed method outperformed the other two in the three directions most of the time. Then, the true positions of the test points were connected as a virtual trajectory and compared with the trajectories connected by positioning results, as shown in Figure 12. The total travel distance of the trajectory at this site was 828.66 m. The plane location error was small with good GPS availability and increased when confronted with satellite loss. At most points, the plane location error of the proposed method was smaller than that of the GPS-only and the GPS + DTMB method, which is consistent with the results in Figure 11.

**Figure 13:**
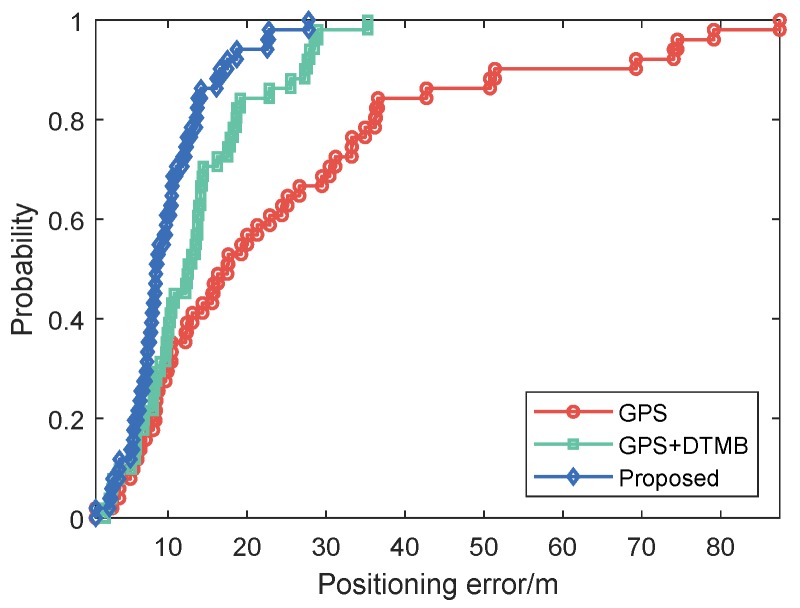
Empirical CDF curves of GPS-only, GPS + DTMB and the proposed method at Site 1.

**Figure 14:**
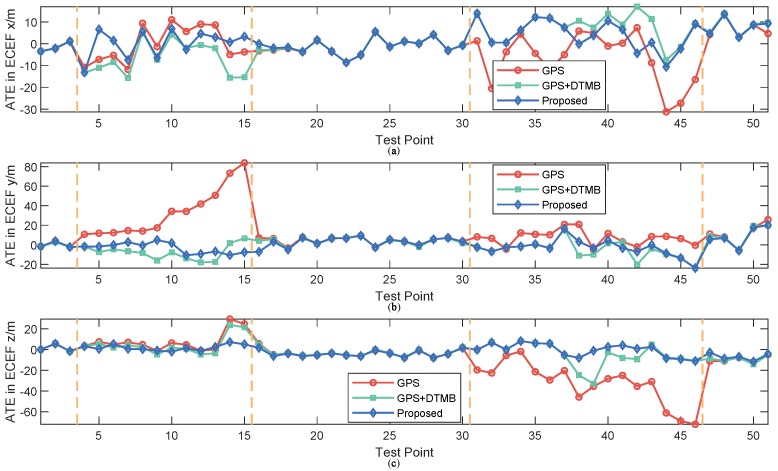
ATE of the three methods at each test point at Site 1: (**a**) ATE in *x* axis direction of the ECEF system; (**b**) ATE in *y* axis direction of the ECEF system; and (**c**) ATE in *z* axis direction of the ECEF system.

**Figure 15:**
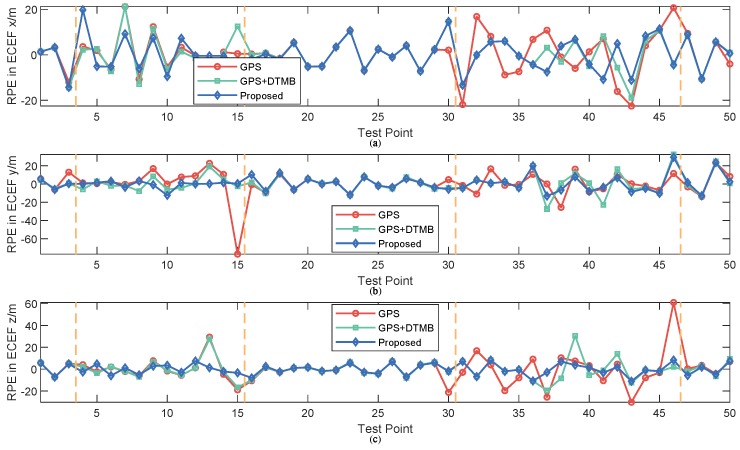
RPE of the three methods at each test point at Site 1: (**a**) RPE in *x* axis direction of the ECEF system; (**b**) RPE in *y* axis direction of the ECEF system; and (**c**) RPE in *z* axis direction of the ECEF system.

For better evaluation of error performance of the proposed method, empirical cumulative distribution functions (CDF) of positioning errors of the three methods are shown in Figure 13. The 1σ, 2σ and 3σ errors are also included in Table 3, where we can see that 3σ error of the proposed method was 21.27% less than that of GPS + DTMB and 68.21% less than that of GPS. We also calculated the absolute trajectory errors (ATE) and relative pose errors (RPE) [29] of the three methods at this site, as shown in Figure 14 and Figure 15. The ECEF coordinate system was chosen as the reference coordinate system to calculate ATE and RPE. The time interval Δ was chosen as 1 second in the calculation of RPE. In the figures, we can see that the proposed method achieved the best performance among the three methods most of the time considering both ATE and RPE. We also calculated the average value and root mean square (RMSE) value of ATE and RPE (Table 4). It can be seen that mean ATE of the proposed method was 59.62% less than that of GPS-only and 26.83% less than that of GPS + DTMB, and for RMSE of ATE the improvement was, correspondingly, 65.39% and 27.87%. On the other hand, mean RPE of the proposed method was 34.26% less than that of GPS-only and 18.39% less than that of GPS + DTMB, and for RMSE of RPE the improvement was, correspondingly, 43.42% and 22.22%.

**Figure 16:**
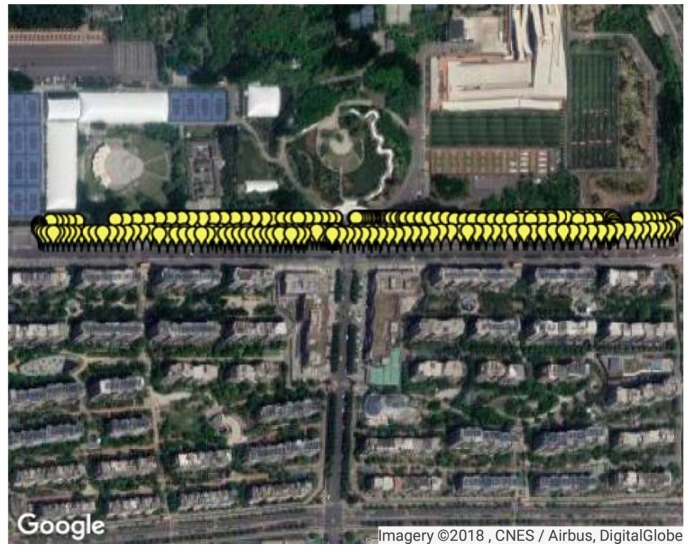
The second test site and test points. The yellow markers on the map denote the test points.

The second test site was on a two-way road, where the single-trip length was around 850 m. Data collection was performed on a car at the frequency of 1 Hz and the total travel distance of test data was 3179.95 m, as shown in Figure 16. FM fingerprint data were collected along the test area. There were 361 test points and 395 reference points at Site 2. In consideration of the density of RPs here, interpolation was not performed at Site 2.

**Figure 17:**
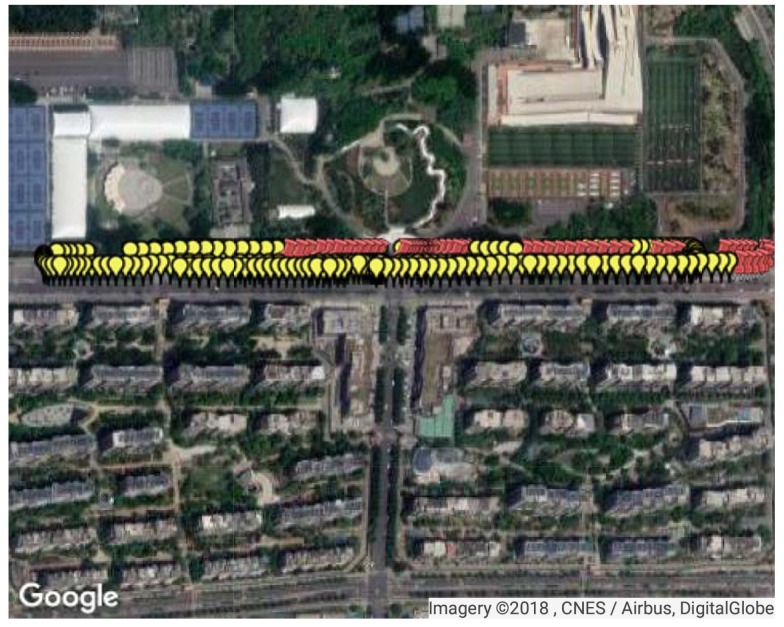
Illustration of points with and without manual satellite loss at Site 2. The red pushpins are points with satellite loss.

**Figure 18:**
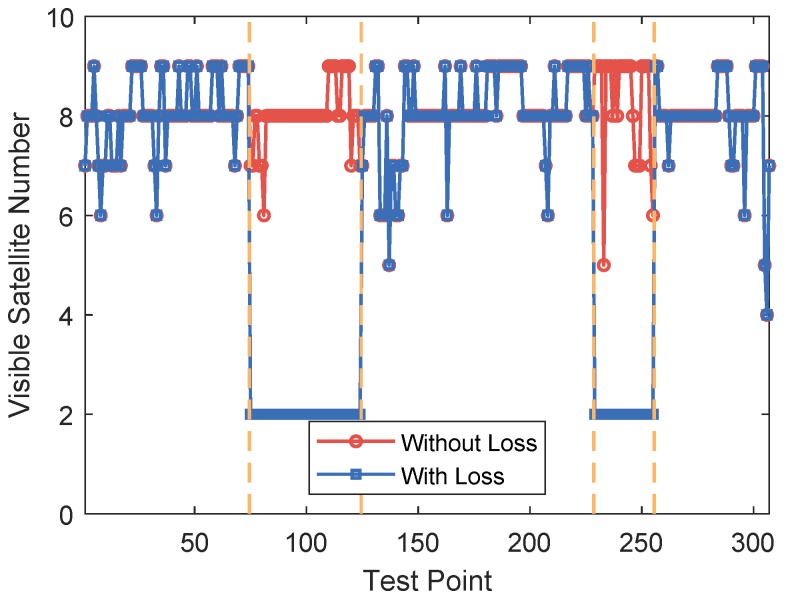
Number of available satellites at each test point at Site 2.

**Figure 19:**
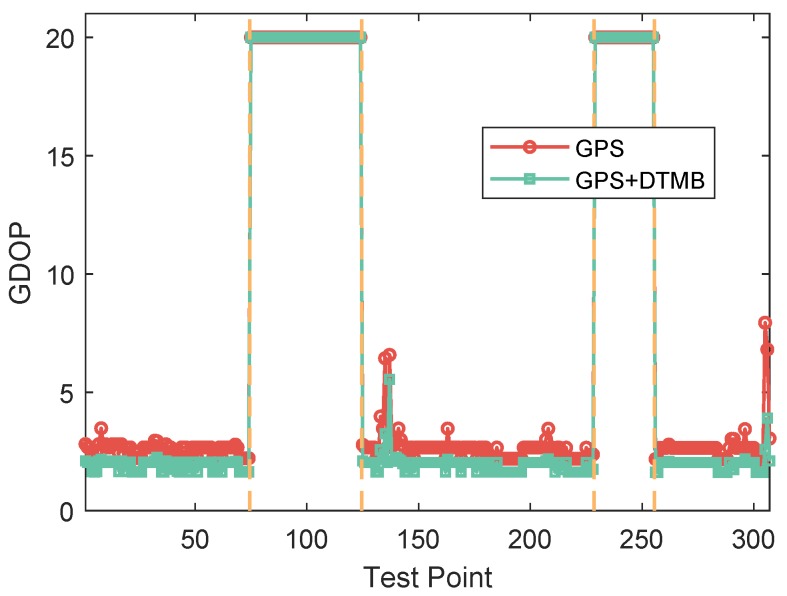
GDOP of GPS and GPS + DTMB at each test point at Site 2.

**Figure 20:**
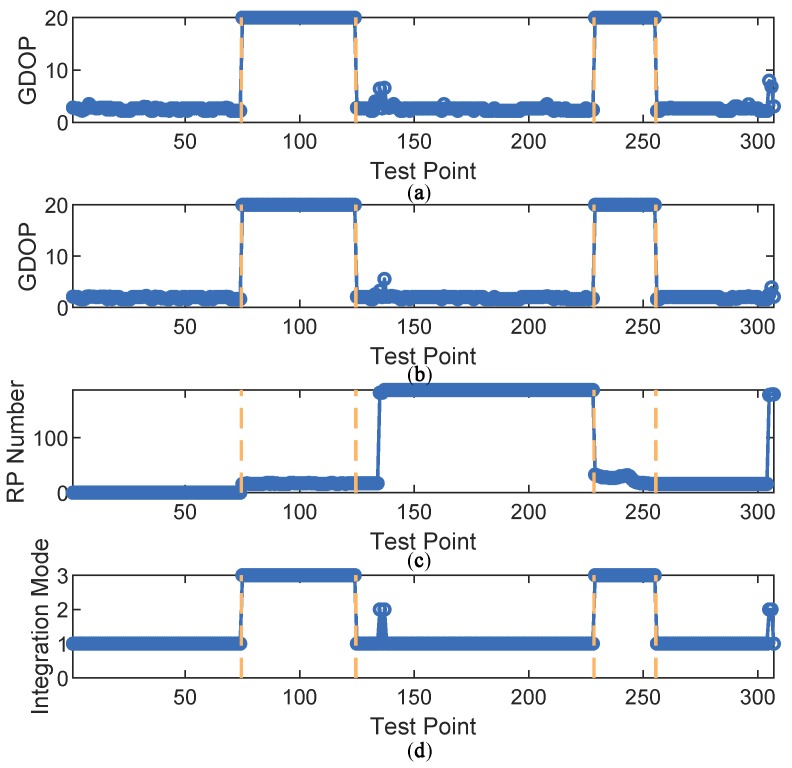
The inputs and output of the fuzzy inference system at Site 2: (**a**) GDOP of GPS-only mode; (**b**) GDOP of GPS + DTMB; (**c**) the number of RPs within constraint range; and (**d**) integration Mode.

Two segments were chosen as the areas with satellite loss at Site 2, where available satellite number was restricted to two. Test points at the two segments are shown on satellite map as the red points in Figure 17. Visible satellite numbers at the sampling points before and after manual satellite signal shielding are shown as Figure 18. In the figure, we can see that before manual satellite loss there were more than five available satellite at most test points. Restriction on satellite number led to changes in GDOP, as shown in Figure 19. When signals from some satellites were shielded, GDOP increased instantly. The inputs and output of our fuzzy inference system are shown in Figure 20. In the figure, we can see that the integration modes at points without satellite loss were mostly GPS-only and at points with satellite loss remained the proposed GPS + DTMB + FM mode.

**Figure 21:**
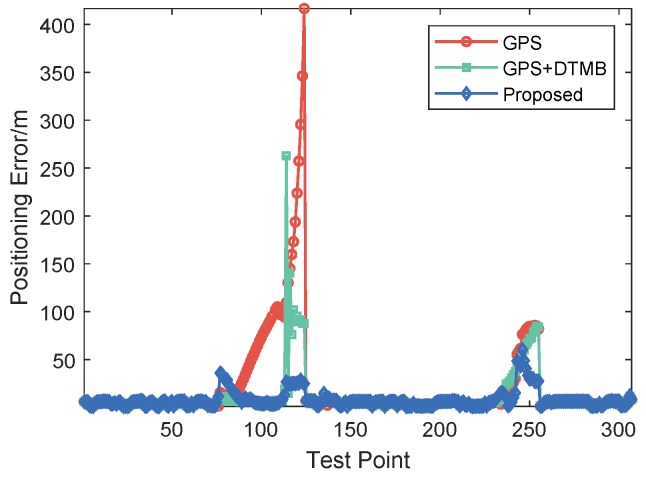
Positioning error at each test point at Site 2.

**Figure 22:**
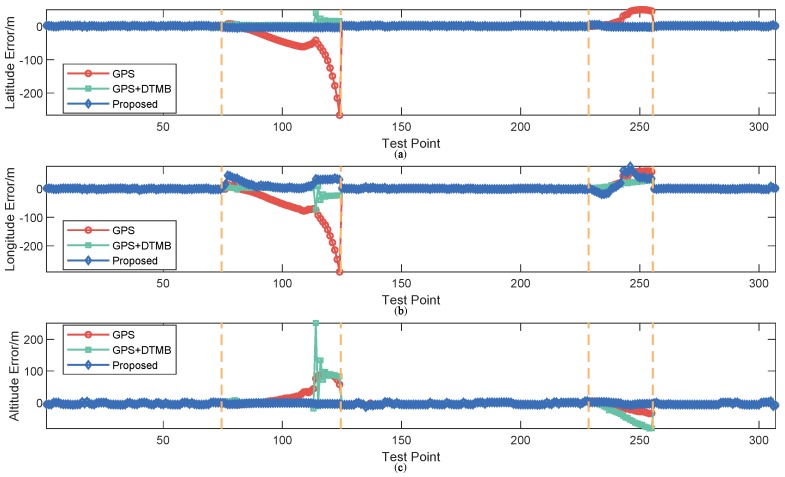
Positioning error of each point in latitude/longitude/altitude direction at Site 2: (**a**) error in latitude direction; (**b**) error in longitude direction; and (**c**) error in altitude direction.

**Figure 23:**
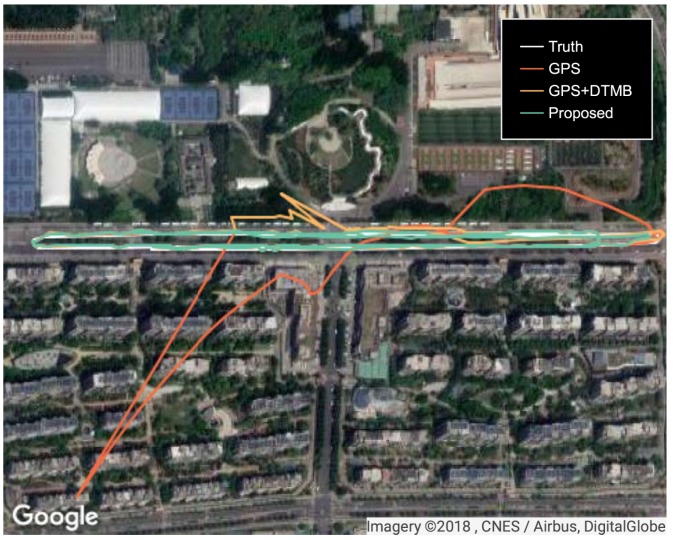
Trajectories generated from true positions and positioning results at Site 2.

Positioning errors of the three approaches at Site 2 are shown as Figure 21, where it is shown that the overall error of the proposed method was the smallest of the three. At the first segment with satellite loss, the error of GPS increased rapidly due to lack of sufficient satellites. Although not large at first, the error of GPS + DTMB also amounted gradually. As for the proposed method, its error was always constrained within 36 m. At the second segment with satellite loss, the relative relationship of positioning error of the three methods was similar to that of the first segment. Errors in latitude/longitude/altitude direction of the three methods are shown in Figure 22, and trajectories generated by the three methods are shown in Figure 23. In the two figures, we can see that positioning accuracy of the proposed method outperformed those of the other two methods in the three directions most of the time.

**Figure 24:**
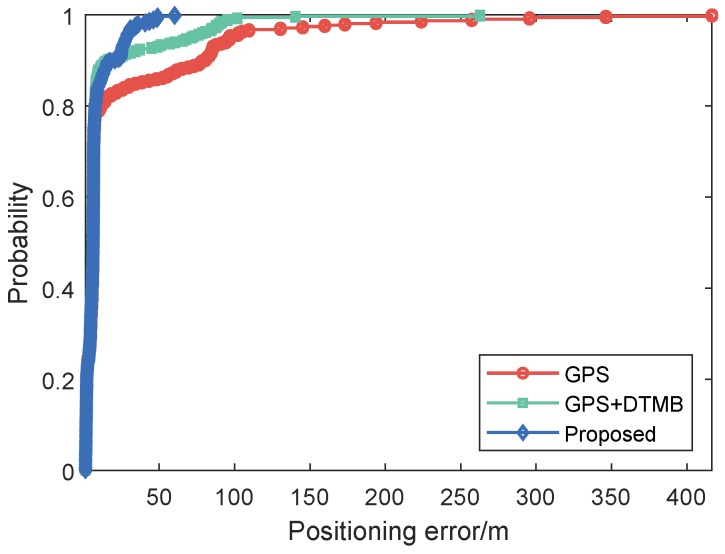
Empirical CDF curves of GPS-only, GPS + DTMB and the proposed method at Site 2.

**Figure 25:**
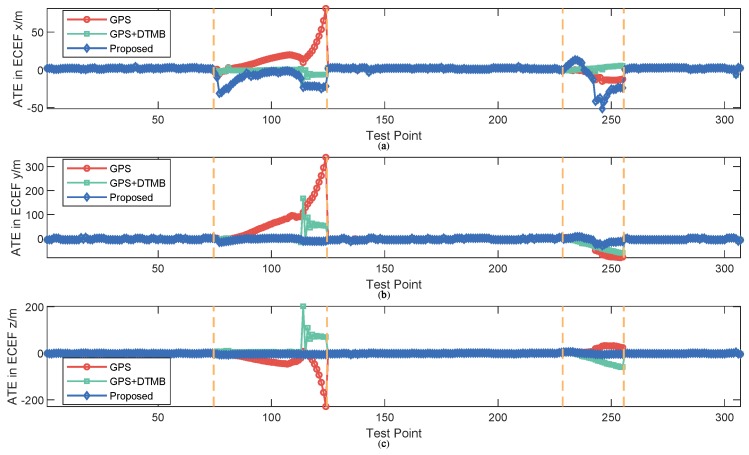
ATE of the three methods at each test point at Site 2: (**a**) ATE in *x* axis direction of the ECEF system; (**b**) ATE in *y* axis direction of the ECEF system; and (**c**) ATE in *z* axis direction of the ECEF system.

**Figure 26:**
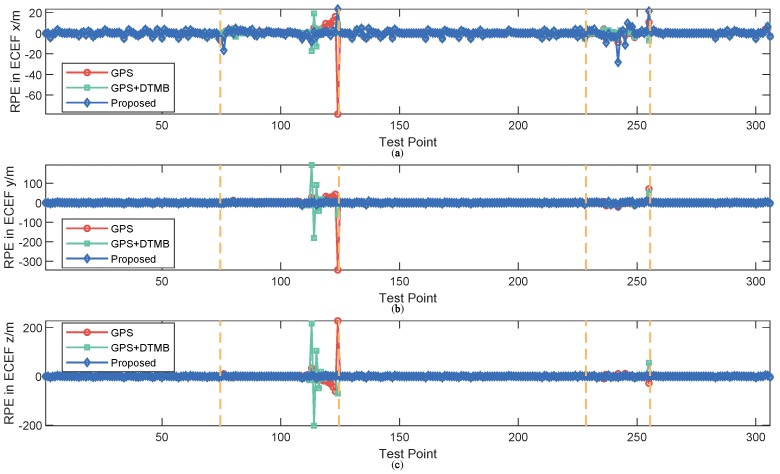
RPE of the three methods at each test point at Site 2: (**a**) RPE in *x* axis direction of the ECEF system; (**b**) RPE in *y* axis direction of the ECEF system; and (**c**) RPE in *z* axis direction of the ECEF system.

Empirical CDF of positioning errors of the three methods are illustrated as Figure 24, and 1σ, 2σ and 3σ errors are given in Table 5. We can see in the figure and the table that the proposed method outperfomed GPS and GPS + DTMB in overall positioning accuracy. The 3σ error improvement of the proposed method was 85.54% compared to the GPS-only method and 77.08% compared to the GPS + DTMB method. The absolute trajectory errors (ATE) and relative pose errors (RPE) of the three methods at Site 2 are shown in Figure 25 and Figure 26. In the two figures, we can see that the proposed method outperformed the other two methods most of the time considering both ATE and RPE. Although the proposed method showed larger ATE error in ECEF *x* direction at the beginning of the first segment with satellite loss, the positioning result converged in a short time and kept good accuracy afterwards. We also calculated the average value and root mean square (RMSE) value of ATE and RPE (Table 6). It can be seen in the table that mean ATE of the proposed method was 63.52% less than that of GPS-only and 33.11% less than that of GPS + DTMB, and for RMSE ATE the improvement was, correspondingly, 77.78% and 56.55%. On the other hand, mean RPE of the proposed method was 49.91% less than that of GPS-only and 50.88% less than that of GPS + DTMB, and for RMSE RPE the improvement was, correspondingly, 81.33% and 81.21%. From the analysis above, it can be concluded that positioning error of GPS + DTMB was smaller than that of GPS, and the proposed method can further improve positioning accuracy with the aid of FM information and adaptive integration mode selection.

## 7. Conclusions

In this paper, an outdoor positioning method with integration of the GPS/DTMB/FM signals and adaptive integration mode selection is proposed and its error performance is presented. On the one hand, DTMB is introduced as information supplement for GPS positioning when faced with satellite shortage, and FM is also utilized to cooperate with GPS for positioning accuracy improvement. On the other hand, a fuzzy inference system is designed to determine the most appropriate integration mode of GPS, DTMB and FM signals based on indicators of environment condition. Experimental results indicate that the proposed method can make reasonable choice on integration mode according to environment conditions when confronted with shortage of available GPS satellites and yield more accurate positioning results than the GPS-only and GPS + DTMB method.

## Figures and Tables

**Table 1:** Symbols for integration modes.

Integration Mode	Symbol
GPS only	$Y^{A}$
GPS + DTMB	$Y^{B}$
GPS + DTMB + FM	$Y^{C}$

**Table 2:** Fuzzy rules in our system.

$x_{1}$	$x_{2}$	$X_{2}^{S}$		$X_{2}^{M}$		$X_{2}^{L}$	
	$x_{3}$	$X_{3}^{S}$	$X_{3}^{L}$	$X_{3}^{S}$	$X_{3}^{L}$	$X_{3}^{S}$	$X_{3}^{L}$
$X_{1}^{S}$		$Y^{A}$	$Y^{A}$	$Y^{A}$	$Y^{A}$	$Y^{A}$	$Y^{A}$
$X_{1}^{L}$		$Y^{A}$	$Y^{A}$	$Y^{B}$	$Y^{C}$	$Y^{C}$	$Y^{C}$

**Table 3:** Errors of different positioning methods at Site 1.

Positioning Method	1σ Error/m	2σ Error/m	3σ Error/m
GPS	29.49	74.51	87.46
GPS + DTMB	14.46	28.50	35.31
Proposed	10.56	22.57	27.80

**Table 4:** Mean and RMSE values of ATE and RPE of different methods at Site 1.

Positioning Method	Mean ATE/m	RMSE ATE/m	Mean RPE/m	RMSE RPE/m
GPS	24.52	32.45	16.61	21.72
GPS + DTMB	13.53	15.57	13.38	15.80
Proposed	9.90	11.23	10.92	12.29

**Table 5:** Errors of different positioning methods at Site 2.

Positioning Method	1σ Error/m	2σ Error/m	3σ Error/m
GPS	6.47	96.89	416.60
GPS + DTMB	6.45	71.31	262.80
Proposed	6.42	28.23	60.23

**Table 6:** Mean and RMSE value of ATE and RPE of different methods at Site 2.

Positioning Method	Mean ATE/m	RMSE ATE/m	Mean RPE/m	RMSE RPE/m
GPS	21.60	53.42	5.57	25.93
GPS + DTMB	11.78	27.32	5.68	25.76
Proposed	7.88	11.87	2.79	4.84
